# MfbHLH38, a *Myrothamnus flabellifolia* bHLH transcription factor, confers tolerance to drought and salinity stresses in *Arabidopsis*

**DOI:** 10.1186/s12870-020-02732-6

**Published:** 2020-12-02

**Authors:** Jia-Rui Qiu, Zhuo Huang, Xiang-Ying Xiang, Wen-Xin Xu, Jia-Tong Wang, Jia Chen, Li Song, Yao Xiao, Xi Li, Jun Ma, Shi-Zhen Cai, Ling-Xia Sun, Cai-Zhong Jiang

**Affiliations:** 1 College of Landscape Architecture, Sichuan Agricultural University, Wenjiang, 611130 Sichuan China; 2 Department of Plant Sciences, University of California Davis, Davis, CA 95616 USA; 3 Crops Pathology and Genetics Research Unit, United States Department of Agriculture, Agricultural Research Service, Davis, CA 95616 USA

**Keywords:** bHLH transcription factor, Abiotic stress tolerance, Abscisic acid (ABA), *Myrothamnus flabellifolia*

## Abstract

**Background:**

The basic helix-loop-helix (bHLH) proteins, a large transcription factors family, are involved in plant growth and development, and defensive response to various environmental stresses. The resurrection plant *Myrothamnus flabellifolia* is known for its extremely strong drought tolerance, but few bHLHs taking part in abiotic stress response have been unveiled in *M. flabellifolia*.

**Results:**

In the present research, we cloned and characterized a dehydration-inducible gene, *MfbHLH38,* from *M. flabellifolia*. The MfbHLH38 protein is localized in the nucleus, where it may act as a transcription factor. Heterologous expression of *MfbHLH38* in *Arabidopsis* improved the tolerance to drought and salinity stresses, as determined by the studies on physiological indexes, such as contents of chlorophyll, malondialdehyde (MDA), proline (Pro), soluble protein, and soluble sugar, water loss rate of detached leaves, reactive oxygen species (ROS) accumulation, as well as antioxidant enzyme activities. Besides, *MfbHLH38* overexpression increased the sensitivity of stomatal closure to mannitol and abscisic acid (ABA), improved ABA level under drought stress, and elevated the expression of genes associated with ABA biosynthesis and ABA responding, sucha as *NCED3*, *P5CS*, and *RD29A*.

**Conclusions:**

Our results presented evidence that *MfbHLH38* enhanced tolerance to drought and salinity stresses in *Arabidopsis* through increasing water retention ability, regulating osmotic balance, decreasing stress-induced oxidation damage, and possibly participated in ABA-dependent stress-responding pathway.

## Background

Plants, as sessile species, are vulnerable to changing environmental conditions, and the increasing drought and salinity stresses usually restrict the development and growth of plants through disturbing ion homeostasis, reducing nutrient uptake, and exacerbating oxidation stress [1]. To adapt to these disadvantaged conditions of environmental stresses, plants have formed a variety of complex coping mechanisms during the evolution process. Signal transduction and transcription regulation play important roles in the sophisticated biochemistry and molecular regulatory networks when plants replying to different stresses. Abscisic acid (ABA) as a ubiquitous plant hormone is involved in the network of stress signaling responding to environmental stimulation and plays an irreplaceable part in diverse biological processes of plants under biotic and abiotic stress conditions [2, 3]. Elevated ABA levels can activate certain transcription factors (TFs), thereby regulating the expression of diverse downstream genes [4]. Hitherto, a range of stress-responsive TFs have been reported with regard to resisting abiotic stress tolerance in different plants [5, 6].

In the plant kingdom, the basic helix-loop-helix (bHLH) transcription factors belong to a large superfamily. It could be subdivided into 26 subsections and participates in multiple transcriptional regulatory pathways [7]. The first bHLH transcription factor, regulatory gene *R*, was isolated from maize, and there are 167 and 177 members in *Arabidopsis* and rice, respectively [8–10]. The bHLH transcription factors are characterized by a highly conserved bHLH domain, comprising of a basic region at its N-terminus and an HLH region following closely. These two regions function as DNA binding and promoting protein-protein interactions, respectively [11, 12]. The core DNA sequence element of target genes recognized by the bHLH proteins is a consensus motif called the E-box (5′-CANNTG-3′), with the palindromic G-box (5′-CACGTG-3′) being one of the most common forms [13, 14].

In recent years, increasing evidence was found that bHLHs are involved in multiple abiotic stress responses by the ABA signal transduction pathway in plants. *AtbHLH92* [15], *AtbHLH17* (*AtAIB*) [16, 17], and *AtbHLH122* [16] regulated response to drought, salinity, osmotic, oxidative or cold stress through an ABA-dependent pathway. Grape *VvbHLH1* endowed transgenic *Arabidopsis* with improved tolerance to salinity and drought via ABA signal network [18]. Chrysanthemum *CmbHLH1* can promote iron absorption through upregulating the expression of Fe-deficiency-responsive genes, and in which ABA may play a key role [19].

bHLH proteins of group Ib, such as bHLH38, bHLH39, bHLH100, and bHLH101, are well known to be involved in regulating iron homeostasis by interacted with FE-DEFICIENCY INDUCED TRANSCRIPTION FACTOR (FIT) (bHLH29), and their expression is strongly induced by iron starvation [20]. They could be regulated by *AtMYC2* [20], which functions as a transcriptional activator in ABA-inducible gene expression under drought stress in *Arabidopsis* [21]*.* The ABRE element (abscisic acid response element), usually found in the promoter region of ABA-inducible genes, was detected in the promoters of the co-expressed genes *AtbHLH039* and *AtbHLH101* [22]*.* Kurt and Filiz also found ABRE elements in the promoter region of bHLH38/39/100/101 gene in *Arabidopsis*, rice, soybean, tomato, and maize [23]. These results suggested that these group Ib bHLH proteins may participate in ABA-response pathways. However, it is still unclear if they take part in response to other abiotic stresses, such as drought and salinity.

Woody resurrection plant *Myrothamnus flabellifolia* Welw. is a unique dwarf shrub worldwide and grows in poor rock conditions [24, 25]. A long period of evolution, as well as mighty adaptability to extreme drought surroundings, make *M. flabellifolia* develop a powerful survival strategy including a well-developed root system and the capability to recover from dehydration [26–28]. Based on the previous study, there are a variety of TFs that play a part in the transcriptional regulatory networks during the dehydration process in *M. flabellifolia*, in which *MfbHLH38* was obviously up-regulated in initial period of dehydrating treatment [29]. The strong stress resistance mechanism of the resurrection plant is inseparable from whose multiple adversity genes. In this study, the *MfbHLH38* was cloned from *M. flabellifolia*. The sequence analysis and functional characterization were performed. Its roles to enhance drought and salinity tolerance were determined and the underlying mechanisms were preliminarily investigated and discussed.

## Results

### Cloning and sequence analysis of *MfbHLH38*

**Fig. 1:**
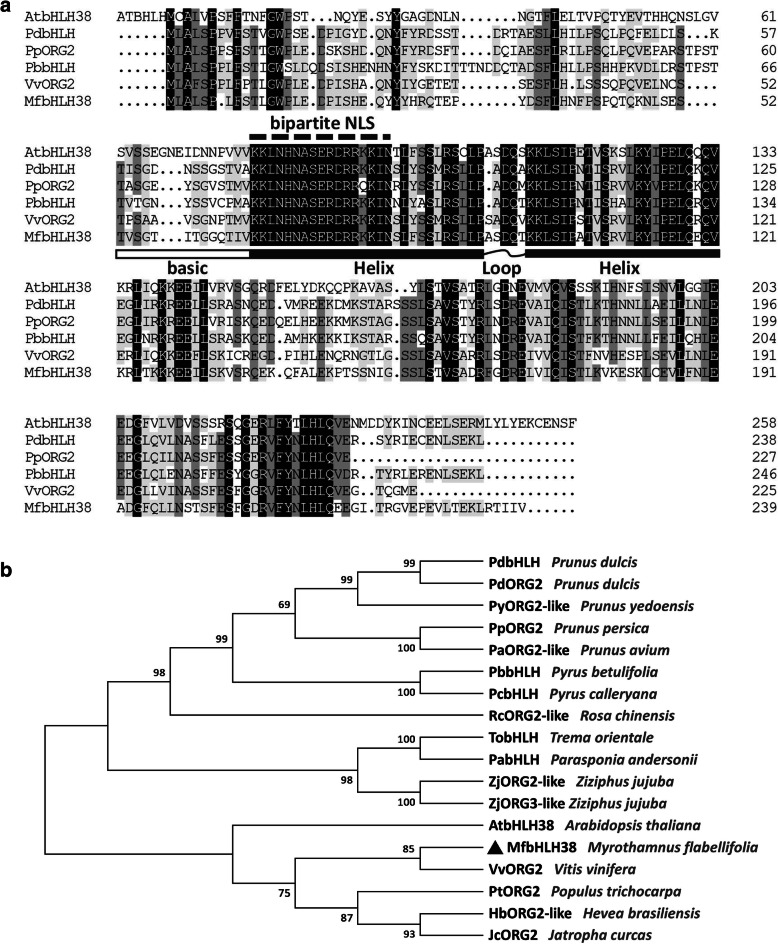
Multiple sequence alignment (**a**) and phylogenetic analysis (**b**) of MfbHLH38 and several highly homologous bHLH proteins. Black and gray shade showed identical and similar amino acids, respectively. Amino acids marked by the dashed line was the deduced NLS. The basic region was marked by the white box, and the curve-linked black boxes indicated the conserved HLH domain. Phylogenetic reconstruction using the neighbor-joining method. The accession numbers for the sequences used are as follows: AtbHLH38 (AT3G56970.1) from *Arabidopsis thaliana*; VvORG2 (RVW89141.1) from *Vitis vinifera*; PdbHLH (BBH07182.1) from *Prunus dulcis*; PpORG2 (XP_020423445.1) from *Prunus persica*; PyORG2-like (PQM37255.1) from *Prunus yedoensis var. nudiflora*; PaORG2-like (XP_021822000.1) from *Prunus avium*; TobHLH (PON88894.1) from *Trema orientale*; PbbHLH (AMX27896.1) from *Pyrus betulifolia*; PcbHLH (AMX27897.1) from *Pyrus calleryana*; ZjORG2-like (XP_024922954.1) from *Ziziphus jujuba*; HbORG2-like (XP_021669234.1) from *Hevea brasiliensis*; RcORG2-like (XP_024162921.1) from *Rosa chinensis*; PabHLH (PON38640.1) from *Parasponia andersonii*; ZjORG3-like (XP_015900576.1) from *Ziziphus jujuba*; PtORG2 (XP_002307969.3) from *Populus trichocarpa*; JcORG2 (XP_012072671.1) from *Jatropha curcas*; and PdORG2 (BBH07187.1) from *Prunus dulcis*

Using PCR amplification, the cDNA sequence of *MfbHLH38* was obtained from *M. flabellifolia*. The obtained sequence is 720 bp in length and encodes an putative protein of 239 amino acids. The theoretical isoelectric point of *MfbHLH38* is 8.84 and the predicted molecular mass is 27.15 kDa. In silico predication detected a a typical bHLH domain and putative bipartite nuclear localization signal (NLS) of “KKLNHNASERDRRKKIN” (Fig. 1a). Multiple alignment of deduced amino acids of MfbHLH38 and several highly homologous bHLH proteins showed a conserved basic region followed by an HLH domain (Fig. 1a). We further performed phylogenetic analysis and found that the MfbHLH38 was most homologous to VvORG2, PtORG2, HbORG2-like, JcORG2 (ORG2 also named bHLH38), and AtbHLH38 which are from *Vitis vinifera*, *Populus trichocarpa*, *Hevea brasiliensis*, *Jatropha curcas*, and *Arabidopsis thaliana*, respectively. (Fig. 1b).

### MfbHLH38 is localized in the nucleus

**Fig. 2:**
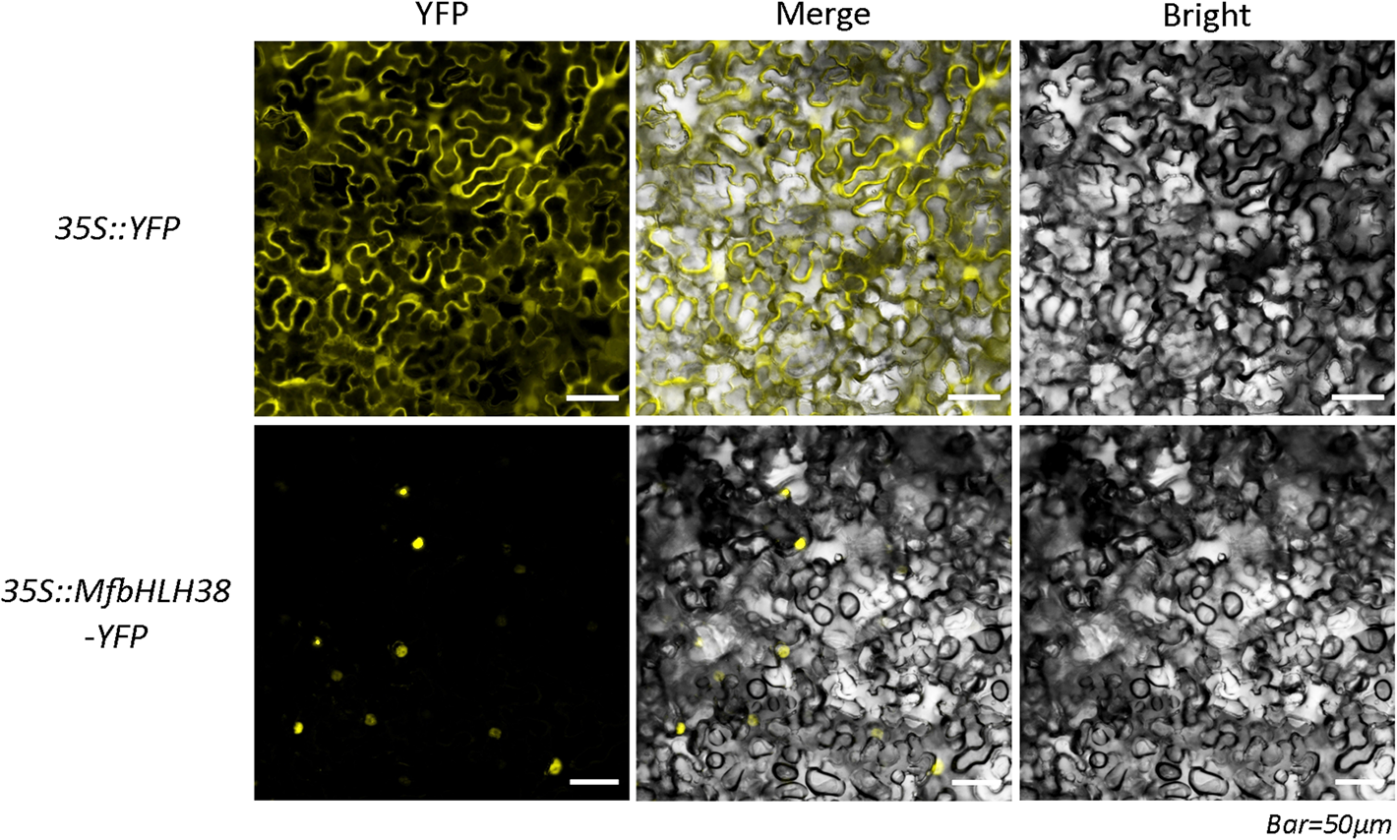
Subcellular localization of MfbHLH38

The predicted NLS in MfbHLH38 (Fig. 1a) suggested that it may function in the nucleus. To confirm this speculation, we performed transient expression of the 35S::MfbHLH38-YFP into leaf epidermal cells of tobacco. Observation using confocal microscope showed that the fluorescence could be detected in the whole cell of 35S::YFP, whereas the intense yellow fluorescence was specifically appeared in the nucleus of 35S:: MfbHLH38-YFP transformed cell. These results proved that MfbHLH38 is located in the nucleus (Fig. 2).

### Overexpressing *MfbHLH38* increased drought and salt tolerance

**Fig. 3:**
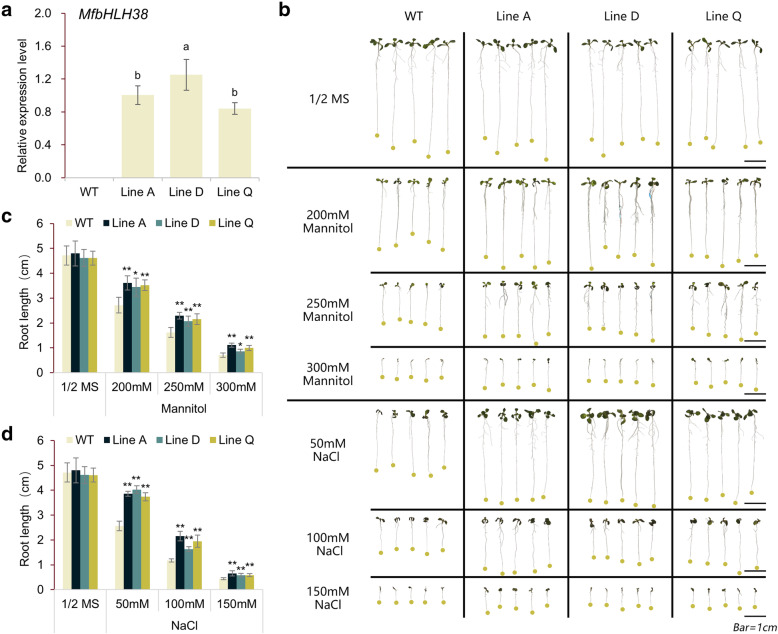
Analysis of drought and salinity tolerance at the seedling stage. (**a**) qRT-PCR analysis of *MfbHLH38* in transgenic plants. The different lowercase letters above the bar showed that the expression abundance was significantly different at *p* < 0.05. (**b**) Morphology of transgenic and WT seedlings growing for nine days on 1/2 MS medium with varying contents of mannitol and NaCl. The primary root length of corresponding plants were measured and analyzed as showed in (**c**) and (**d**). Data are presented as mean and SD values of three independent experiments. Asterisks indicated significant difference (* *P* < 0.05, ** *P* < 0.01, by Independent sample T-test) comparing to WT.

To analyze the potential roles in response to abiotic stress, the *MfbHLH38* was introduced into *Arabidopsis* driven by 35S promotors. T_1_ transgenic *Arabidopsis* lines that overexpressing the *MfbHLH38* gene was acquired from kanamycin resistance screening, and three homozygous T_3_ transgenic lines were randomly selected and used for further analysis. The qRT-PCR analysis indicated that that expression level of *MfbHLH38* could be detected in all three selected transgenic lines, in which Line D exhibited significantly higher expression level than the other two lines (Fig. 3A).

In order to verify whether *MfbHLH38* is associated with drought and salinity stress tolerance, WT and transgenic lines were subjected to stress treatments at both the seedling and adult stages. At the seedling stage, there was no significant difference between wild type and transgenic plants under normal conditions (Fig. 3B). Under treatments of mannitol and salt, transgenic lines exhibited significantly longer roots. This difference was more obvious when rather moderate concentrations of mannitol (250 mM) and NaCl (100 mM) were used (Fig. 3B, C, and D). Consistently, larger leaf area was also found in transgenic lines compared with WT (Fig. 3B).

**Fig. 4:**
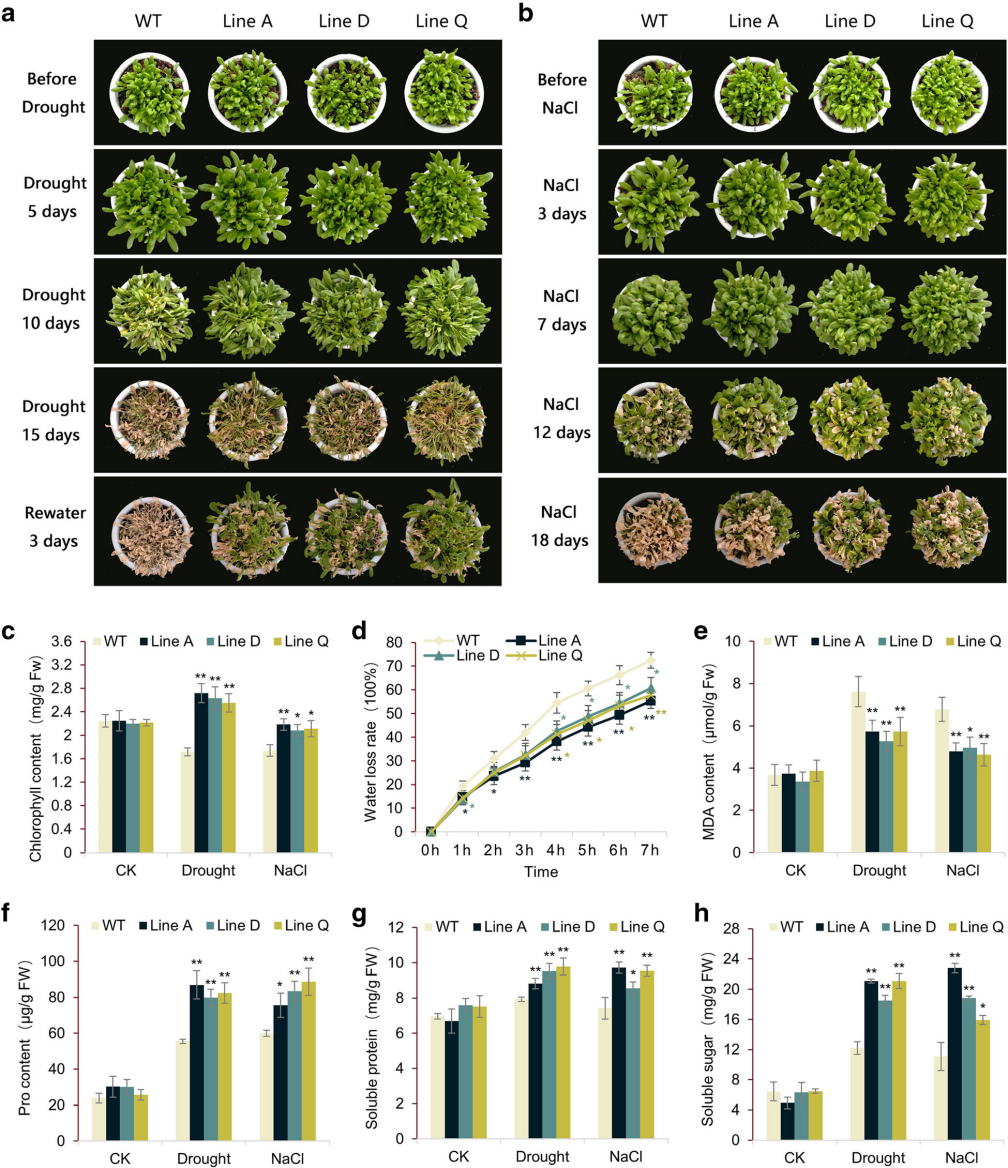
Analysis of drought and salinity tolerance at the adult stage. **a** and **b** showed the growth status of transgenic and WT plants during drought and salinity treatments. **c**-**h** showed measurements of tolerance-related physiological indexes. Data are presented as mean and SD values of three independent experiments. Asterisks indicated significant difference (* *P* < 0.05, ** *P* < 0.01, by Independent sample T-test) comparing to WT.

At the adult stage, treatments of natural drought and 300 mM NaCl were performed on four week old transgenic and WT plants growing in soil. The morphological difference was not remarkable among the transgenic and WT plants before and at early stages of two treatments (Fig. 4). Withholding watering (DAW) for 10 days, the wilting degree of the WT leaves was significantly higher than that of the transgenic lines, and the leaf chlorophyll content of later was 1.48–1.58 times higher than that of former (Fig. 4a, c). At 15 DAW, the WT leaves were basically withered, while a considerable number of leaves on transgenic plants remained light green (Fig. 4a). Three days after re-watering, transgenic plants were partially restored, however, the almost all of the WT plants were dead (Fig. 4a).

The salinity stress negatively influence the plant growth, which was visible about seven days of salt treatment (Fig. 4b), and the leaf chlorophyll content of *MfbHLH38* transgenic lines was 1.20–1.26 times higher than that of WT (Fig. 4c). Twelve days after exposure to salinity stress, more withered leaves were appeared on WT plants comparing to those of transgenic lines. After 18 days, approximately more than 1/3 of transgenic plants stayed green and flowered, whereas almost all leaves of WT were withered (Fig. 4b).

The dynamic water loss rate (WLR) of detached leaves were measured during dehydration. As shown in Fig. 4d, WLR of transgenic plants were significantly lower than that of WT at all-time points except for 0 h, indicating that overexpression of *MfbHLH38* slowed down the water loss. Malondialdehyde (MDA) can severely damage plant cell membranes, thus degree of MDA accumulation is usually considered as a indicator of membrane-lipid peroxidation. In our experiment, although both the drought and salt treatments elevated the MDA content in either the WT or transgenic plants, it stayed in significantly lower levels than that of WT (Fig. 4e).

We compared the contents of several osmotic adjustment substances, including proline, soluble protein, and soluble sugar, among the transgenic and WT plants before and after treatments. Our results revealed the similar patterns that both the drought and salt stresses enhanced osmolyte accumulation in WT and the transgenic lines, but those in later was significantly higher that that of the former (Fig. 4f-h).

### Effect of *MfbHLH38* overexpression on antioxidant metabolism

**Fig. 5:**
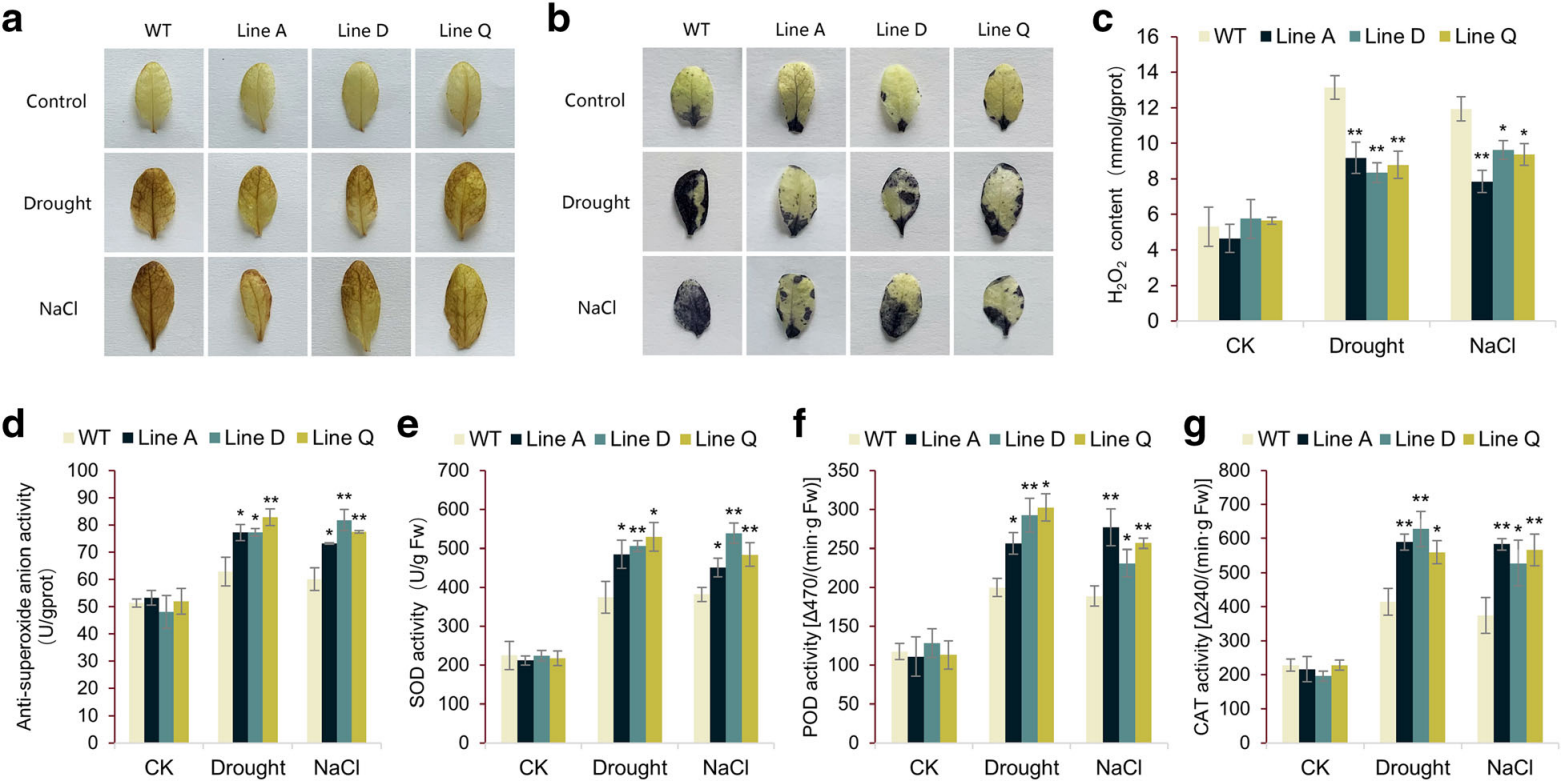
Analysis of ROS accumulation and activities of key antioxidant enzymes under drought and salt treatments. **a** and **b** showed the analysis of H_2_O_2_ and O_2_^−^ accumulation by using histochemical staining with DAB and NBT, respectively. Data are presented as mean and SD values of three independent experiments. Asterisks indicated a significant difference (* *P* < 0.05, ** *P* < 0.01, by Independent sample T-test) comparing to WT.

It is well known that rising of lipid peroxide will aggravate the cellular oxidative damage when plants suffer from the abiotic stresses. This is caused by excessive accumulation of ROS (reactive oxidative species), such as hydrogen peroxide (H_2_O_2_) and superoxide anion radical (O_2_^−^). We employed histochemical staining by 3, 3′-diaminobenzidine (DAB) and nitroblue tetrazolium (NBT) to evaluate cellular ROS content after drought and salt treatments. The WT leaf could be stained in darker color and larger area comparing to those of transgenic plants (Fig. 5a and b), indicating slighter cellular oxidative damage occurred in the transgenic lines. Consistently, less H_2_O_2_ content and higher anti-superoxide anion activity were detected in three transgenic lines (Fig. 5c and d).

**Fig. 6:**
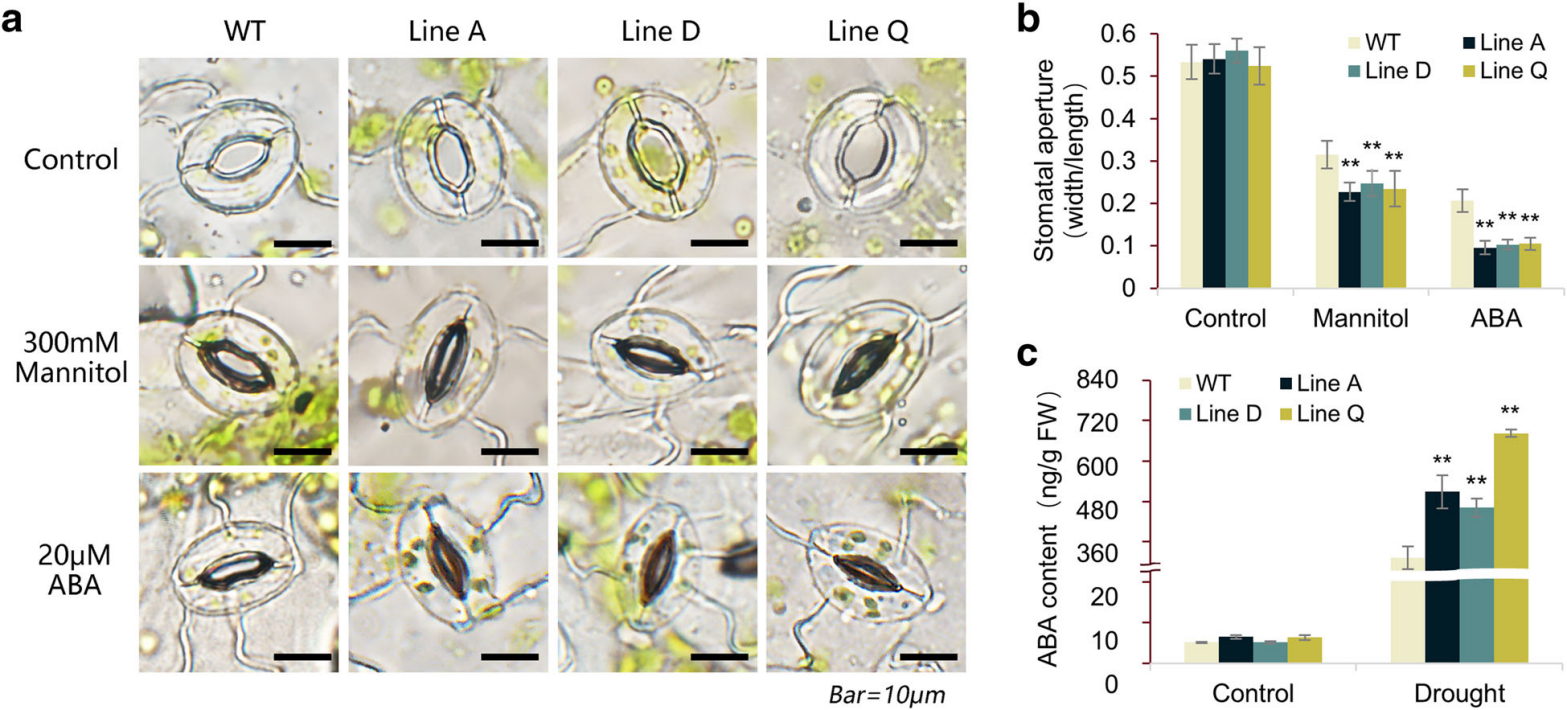
Measurements of Stomatal aperture and endogenous ABA content. **a** Images showed the stomatal aperture of transgenic and WT plants treated by 300 mM mannitol and 20 μM ABA. **b** Changes of the stomatal aperture with or without stress treatment. **c** Quantitative analysis of ABA content in *Arabidopsis* under drought condition. Data are presented as mean and SD values of three independent experiments. Asterisks indicated significant difference (* *P* < 0.05, ** *P* < 0.01, by Independent sample T-test) comparing to WT.

Furthermore, we measured activities of superoxide dismutase (SOD), peroxidase (POD), and catalase (CAT), which are the key enzymes participating in ROS scavenging. Along with the ROS level increasing, the activities of SOD, POD, and CAT were significantly elevated by drought and salt treatments in both the WT and the transgenic plants. However, the enzyme activities of transgenic plants are apparently higher than that of WT (Fig. 6e-g), showing that overexpression of *MfbHLH38* could decrease cellular oxidative damage under stressful conditions through increasing ROS scavenging capacities.

### *MfbHLH38* promoted stomatal closure and the biosynthesis of endogenous ABA

The ABA-mediated stomatal movement plays a central role in transpiration upon water stress. We assessed the stomatal closure under 300 mM mannitol and 20 μM ABA treatments. For both WT and transgenic lines, most of the stomata were open under normal conditions (Fig. 6a), and the stomatal aperture (width/length ratio) of was significantly different between transgenic and WT plants (Fig. 6b). Treatments of mannitol and ABA reduced stomatal aperture of three transgenic lines to 0.23–0.25 and 0.09–0.10, respectively, which were remarkably lower than those of WT plants (0.32 and 0.21) (Fig. 6b). These results demonstrated that *MfbHLH38* promoted stomatal closure in response to mannitol and ABA. We also measured ABA content under drought stress. The accumulation of ABA in WT and transgenic lines increased significantly after drought treatment, however, ABA contents in three transgenic lines were 1.48–2.18 times higher than that of WT plants (Fig. 6c). This result indicated the overexpression of *MfbHLH38* promoted ABA synthesis under drought.

### Overexpression of *MfbHLH38* up-regulated expression levels of ABA-responsive genes

**Fig. 7:**
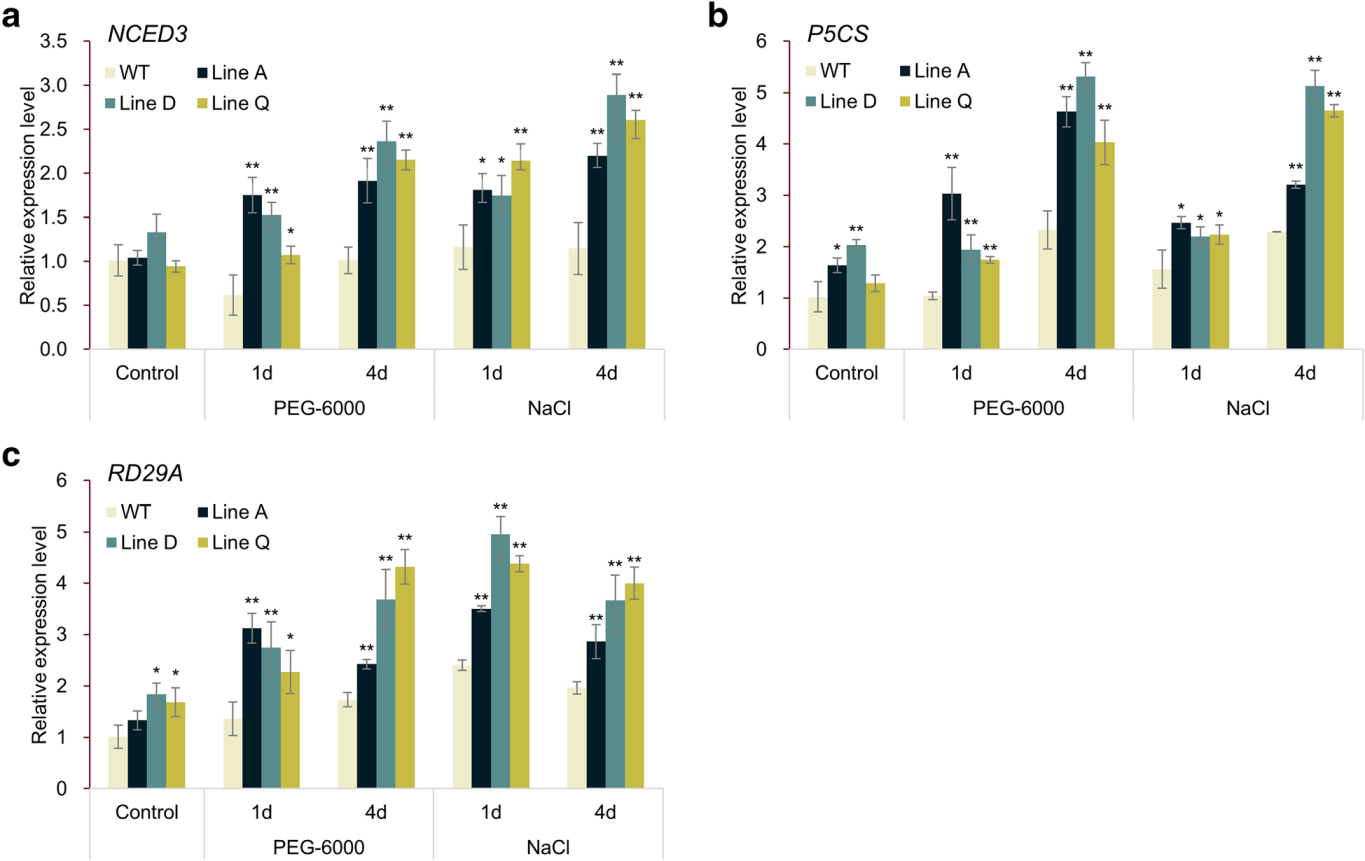
qRT-PCR analysis of relative expression levels of stress-related and ABA-responsive genes. Plants treated by normal condition, 10% PEG-6000, and 300 mM NaCl for one d and four d were analyzed. Data were presented as mean and SD values of three independent experiments. Asterisks indicated significant difference (* *P* < 0.05, ** *P* < 0.01, by Independent sample T-test) comparing to WT.

To further explorer the potential molecular mechanisms underlying enhanced drought and salinity tolerance by *MfbHLH38*-overexpressing, we measured the expression levels of three stress-induced and ABA-responsive genes under artificially simulated drought treatment (10% PEG-6000) and salt treatment (300 mM NaCl) for one day and four days, respectively. As shown in Fig. 7a, the similar expression level of *NCED3* were found among WT and transgenic lines before treatments. After treatments, the expression levels in all three transgenic lines increased faster and higher than that of WT. The *P5CS* and *RD29A* exhibited slightly higher expression levels in transgenic lines before treatments (Fig. 7b and c). Under drought treatment, the expression levels of *P5CS* and *RD29A* in *MfbHLH38*-overexpressing lines were remarkably increased and significantly higher than those of WT plants. Under salinity stress, expression levels of *P5CS* and *RD29A* showed a similar trend of *NCED3*. All these results suggested that *MfbHLH38* positively regulated the expression of ABA-responsive genes in *Arabidopsis* directly or indirectly.

## Discussion

Stress tolerance of plant depends on the adversity genes, and overexpression of these genes can improve the plant’s ability to adapt to a variety of environmental stresses [30]. The function of *bHLH38* in stress tolerance has not yet been exploited, even though its role in maintaining cellular iron homeostasis has been extensively investigated. In the present study, we isolated and identified *MfbHLH38* from *M. flabellifolia.* It contains the high conservative bHLH domain (Fig. 1a), and showed high homology with VvORG2 (*Vitis vinifera*), PtORG2 (*Populus trichocarpa*), HbORG2-like (*Hevea brasiliensis*), and JcORG2 (*Jatropha curcas*) (Fig. 1b).

Further investigation showed that overexpressing *MfbHLH38* in *Arabidopsis* endows it with tolerance to drought and salinity stresses, as revealed by better growth vigor of *MfbHLH38*-overexpressing plants under stress treatments and at either seedling or adult stages (Fig. 3b, 4a and b). The increased adaptability of these transgenic plants to drought and salinity stresses was inseparable from the simultaneous adaptive changes in external morphology and biochemical levels.

Plants can draw water from deep soil through well-developed root systems, thereby improving the efficiency of limited water use under drought conditions [31]. In seedling stress assays, *MfbHLH38* transgenic lines showed stronger growth and longer main roots (Fig. 3c and d). Additionally, plants can reduce water loss in drought conditions by promoting stomatal closure, and ABA-regulated stomatal movement enables plants to improve water retention. For example, stomatal aperture significantly decreased in *PebHLH35*-overexpressing *Arabidopsis* with the increase of drought degree, showing that *PebHLH35* overexpression exhibited good tolerance in transgenic plants to drought stress [32]. In this study, stomatal movements of *MfbHLH38* overexpression lines were more sensitive to mannitol treatment and exogenous ABA (Fig. 6a, b), and exhibited significantly lower water loss rate (Fig. 4d). These results showed that *MfbHLH38* could enhance water uptake and retention, and therefore provides a better water use condition under stress treatments.

Plants can generate osmoregulation substances including proline, soluble proteins as well as soluble sugars under stresses. The accumulations of these osmotic modifiers could assist plants to resist the environmental stresses via maintaining the osmotic equilibrium [33, 34]. Yang et al. found that after drought and salinity stress treatments, the content of proline and soluble sugar in *TabHLH1* transgenic *Arabidopsis* increased, and was significantly higher than that of WT plants [35]. Our data showed that the contents of these three osmoregulation substances were increased obviously upon drought and salinity stresses (Fig. 4f-h). This result was also supported by higher expression levels of *P5CS* under stress in three transgenic lines comparing to WT plants. Thus, *MfbHLH38* may be involved in osmotic regulation directly or indirectly.

MDA content is a measurement of the degree of oxidative stress via reflecting plant membrane-lipid peroxidation level [36]. In this study, less MDA accumulation was detected in *MfbHLH38* transgenic lines (Fig. 4e), suggesting that overexpression of *MfbHLH38* could maintain the membrane-lipid structure stable under drought and salinity conditions. ROS accumulation in plant cells, such as hydrogen peroxide (H_2_O_2_) and superoxide anion (O_2_^−^), promoted by drought and salt stresses leads to irreversible damages to plants [37, 38]. As shown by the results of histochemistry staining with DAB and NBT, and measurements of H_2_O_2_ content and anti-superoxide anion activity, *MfbHLH38* transgenic lines presented a less increase of ROS accumulation under drought and salinity stresses (Fig. 5a-d). Antioxidant enzymes have important functions in reducing oxidative injury to plants caused by drought and salt stresses [39]. Consistent with lower ROS accumulation, overexpression of *MfbHLH38* remarkably increased the activities of antioxidant enzymes, SOD, CAT, and POD, upon stress conditions (Fig. 5e-g), indicating that *MfbHLH38* could enhance the ROS scavenging system and reduced oxidative injury under stress.

As a critical plant hormone, ABA is involved in various developmental processes and stress signaling transduction mechanisms in plants [40]. Several bHLH TFs were reported to induce ABA biosynthesis and are involved in stress tolerance. For example, the grape VvbHLH1 conferred great tolerance for transgenic *Arabidopsis* to drought stress by increasing ABA levels [18]. In the present study, the significantly higher ABA contents in three transgenic lines comparing to WT under drought treatment were found (Fig. 6c). This is evident that overexpression of *MfbHLH38* enhanced ABA biosynthesis, which explained promoted stomatal closure under stress treatments, as well as elevated expression levels of ABA-biosynthesis gene *NCED3* and ABA- and stress-responsive genes *P5CS* and *RD29A* (Fig. 7). These results suggested that *MfbHLH38* might positively function in plant defense via the ABA-dependent pathway.

bHLH38 plays a positive regulatory role in iron deficiency [41]. In this study, we demonstrated that MfbHLH38 also positively regulated drought and salt stress responses. This result suggested that *MfbHLH38* might mediate crosstalk between regulating Fe-deficiency and other abiotic stress responses [42]. As iron acts on the particular active sites of some antioxidant enzymes, for instance, SOD, POD, and CAT [43], overexpressing *MfbHLH38* may help to retain or increase activities of antioxidant enzymes through promoting Fe uptake. Furthermore, iron (Fe) plays an important role in chlorophyll biosynthesis and photosynthesis. However, water deficiency affects transporting nutrients to roots and nutrients utilization ratio, such as micronutrient Fe [44, 45]. And high-concentration sodium also interferes with the uptake and translocation of iron and other mineral elements [46, 47]. Then the stresses initiated Fe deficiency can further cause the damage on chlorophyll, thereby seriously interfere with the photosynthesis process it involved [48]. Babaeian et al. applied Fe fertilizers to the leaves of sunflowers at the flowering and seed filling stages under drought conditions, and higher chlorophyll fluorescence (FV/FM) and chlorophyll content compared to the control plants were detected, proving that the Fe helps to improve photosynthesis in drought stress [49]. Consequently, it is possible that overexpression of *MfbHLH38* may promote Fe uptake under stressful condition and slow down the damage on chlorophyll and its biosynthesis, and hence ensure plants better growth comparing to WT. However, further study is deserved to excavate the exact mechanisms underlying the positive role of MfbHLH38 in regulating abiotic stress response.

## Conclusions

This study reported the characterization of *MfbHLH38* encoding a bHLH transcription factor homologous to AtbHLH38. We demonstrated that heterologous expression of *MfbHLH38* in *Arabidopsis* significantly enhanced tolerance to drought and salinity by increasing water retention ability, regulating osmotic balance, strengthening stress-induced oxidation scavenging system, and possibly participated in ABA-dependent stress-responding pathway. This is the first report of involvement of bHLH38 in drought and salinity stress tolerance regulation. MfbHLH38 may have potential to be utilized in drought and salt stress tolerance improvement in plants. And it will be beneficial to further explore the molecular mechanism underlying the survival of *M. flabellifoli* from the desiccation environment.

## Methods

### Plant materials and growth conditions

The *M. flabellifolia* was originally obtained from Dr. Matthew Opel (University

of Connecticut). *M. flabellifolia* was firstly recorded and named by Welwitsch [50]. One of the voucher specimens of this species could be found in National Museum of Natural History (10th St. & Constitution Ave. NW Washington, D.C. USA) at Botany Collections (US Catalog No.: 2921412 Barcode: 00072109). The plants used in this study were grown in plastic pots under condition of 12 h light/12 h dark at 22 °C/18 °C, 60% relative air humidity and sufficient light .

Seeds of *Arabidopsis* ecotype *Columbia* (Wild-type, WT) and overexpression lines were sterilized using diluted bleach solution for 5 min, and washed with using sterilized deionized water for three times. The sterilized seeds were placed on 1/2-strength Murashige and Skoog (MS) medium containing 0.7% (w/v) agar and 2% (w/v) sucrose and with adjusted pH of 5.8–6.0. After vernalizationat (4 °C for two days), the medium plate was placed in an illuminating incubator for about 10 days. The young seedlings were transplanted into pots filled with cultivation substrate of soil and vermiculite (1:1) in a growth chamber, and then were grown under the long day (16 h light/8 h dark and 24 °C/22 °C) condition and about 75% relative humidity for four weeks before stress treatments.

### Cloning and sequence analysis of *MfbHLH38*

Total RNA was extracted from fresh leaves using Plant Total RNA Isolation Kit (TINAGENE Co., Beijing, China). The first-strand cDNA synthesis was conducted using Reverse Transcriptase M-MLV (RNaseH-) (Takara Bio, Dalian, China) according to the protocol provided by the kit. The coding sequence (CDS) of *MfbHLH38* was amplified by PCR using Phanta Max Super-Fidelity DNA Polymerase (Vazyme Biotech Co., Nanjing, China) with a primer pair, 5′-TCCCCCGGG ATGCTAGCTCTATCTCCTTT-3′ (*Sma*І site is underlined) and 5′-GACTAGTTCATACGATGATGGTACGTA-3′ (*Spe*I site is underlined). The target amplified fragment was recovered from gel and ligated onto the pEasy-T1 Simple vector (TransGen Biotech, Beijing, China). The resulting construct, of pEasy-T1-MfbHLH38, was transformed into *E. coli* strain DH5α and the putative positive clones were confirmed by Sanger sequencing (TsingKe Biotech Co., Beijing, China).

The open reading frame (ORF) was detected by ORFfinder of NCBI (https://www.ncbi.nlm.nih.gov/orffinder/). Isoelectric point (pI) and molecular weight were caculated by ExPASy (https://web.expasy.org/compute_pi/). SMART (http://smart.embl-heidelberg.de/) was used to analyzing potential conserved domains in the deduced protein sequence. Homologs of MfbHLH38 from different species were searched using BLASTP (https://blast.ncbi.nlm.nih.gov/Blast.cgi) against nr database. Multiple alignments and subsequent phylogenetic analyses were performed with MEGA 7.0 [51] using the neighbor-joining method with the bootstrap test of 1000 replicates. The secondary structure of deduced protein was predicted by Jpred 4 (http://www.compbio.dundee.ac.uk/jpred/index.html).

### Subcellular localization of MfbHLH38

The complete ORF without the termination codons of *MfbHLH38* was obtained with primers containing adaptor sequences (italicized) complementary with vector sequence flanking ligation site, forward, 5′- *ACCAGTCTCTCTCTC*AAGCTTATGCTAGCTCTACTATCTCCTTT − 3′ (*Hind*ІІІ site is underlined) and reverse, 5′- *GCTCACCATACTAGT*GGATCCTACGATGATGATGGTACGTA − 3′ (*BamH*І site is underlined). The amplified fragment was double-digested by *BamH*І and *Hind*ІІІ and inserted into the pHB-YFP vector to formed a expression construct MfbHLH38-YFP, which was fused with the gene encoding yellow fluorescent protein (YFP) driven by a CaMV (Cauliflower Mosaic virus) 35S promoter. Both the 35S::MfbHLH38-YFP and 35S::YFP were transformed into the *Agrobacterium tumefaciens* strain *GV3101* by employing the freezing-thawing method, respectively. Leaves of four-week-old (*Nicotiana benthamiana*) wild-type tobacco were injected with *A. tumefaciens.* All the transformed tobacco plant were grown in dark for 16 h at 22 °C and then moved to normal condition for two days before observing the YFP by a laser confocal scanning microscope (Nikon, Tokyo, Japan).

### Plasmid construction transformation in Arabidopsis

The complete coding region of *MfbHLH38* was amplified using gene-specific primers supplied with either a *Sma*І or *Spe*I restriction site. The amplicon was double digested and ligated into the corresponding sites pGSA-1403, The resulting construct 35S::pGSA1403-MfbHLH38 was introduced into the *A. tumefaciens* strain LBA4404, and then transformed into *Arabidopsis* using the floral-dip transformation method [52]. T_0_ seeds were screened on 1/2 MS medium supplying with kanamycin (50 μg/ml). The seedlings resistant to kanamycin were transplanted into pots with soil and the positive transgenic plants were further verified by PCR. Three homozygous positive lines (T_3_) were randomly selected for further experiments.

### Expression analysis of *MfbHLH38* and ABA-responsive genes

The four-week-old seedlings were subjected to normal condition, as well as simulated drought (10% PEG-6000) and salt treatments (300 mM NaCl) for one day and four days, respectively. The leaves sampled from same positions of plants at one day and four days of treatments were used for expression level analysis. Total RNA extraction and the first-strand cDNA synthesis were conducted following the procedures mentioned above. The 25 μl reaction mixture (Innovagene Biotech) was consisted of 12.5 μl of 2 × Taq SYBR Green qPCR Mix, 0.5 μl of each primer (10 μM), 4 μl of diluted cDNA, and 7.5 μl of Nuclease-free H_2_O. The PCR was performed using CFX Connect fluorescent quantitative PCR instrument (Bio-Rad, Hercules, CA, USA), and the amplification condition was as follows: 94 °C for 5 min, 42 cycles of 94 °C for 8 s, and 60 °C for 60s. The *AtActin2* was used as the internal reference and the relative expression levels was calculated with 2^−ΔΔCT^ method. Each qRT-PCR experiment performed as at least three technical and biological replicates. The primers used were for *MfbHLH38*, 5′- TCGGAGAGAGGAAAACAAGC − 3′ (forward) and 5′- TTTTCCTTCACCCCAGACAC − 3′ (reverse); *NCED3*, 5′- CGAGCCGTGGCCTAAAGTCT − 3′ (forward) and 5′- GCTCCGATGAATGTACCGTGAA − 3′ (reverse); *P5CS*, 5′- GGTGGACCAAGGGCAAGTAAGATA − 3′ (forward) and 5′- TCGGAAACCATCTGAGAATCTTGT − 3′ (reverse); *RD29A*, 5′- GATAACGTTGGAGGAAGAGTCGG − 3′ (forward) and 5′- TCCTGATTCACCTGGAAATTTCG − 3′ (reverse); and *AtActin2*, 5′- GGAAGGATCTGTACGGTAAC − 3′ (forward) and 5′- TGTGAACGATTCCTGGACCT − 3′ (reverse).

### Analysis of tolerance to drought and salinity

For stress assays at seedling stage, the sterilized seeds were placed on 1/2 MS solid medium varying in mannitol (0–300 mM) and NaCl (0–150 mM) concentrations. Culture dishes were vertically settled and incubated with a cycle of 16 h/8 h of light (24 °C)/dark (22 °C). Nine days later, the taproot length of each sample (15 seedlings per line for every petri dish) was measured. Each experiment was performed in three replicates.

To explore the drought and salinity tolerance for mature plants, the culture substrate after being fully infiltrated with water is equally divided into each pot. Approximately 50 vernalized (4 °C for two days) seeds of each line were sown into pots under regular cultivation condition. Four-week-old plants were treated by drought and salinity stresses. For the drought treatment, every pot was firstly fully irrigated to ensure a saturated water content. For drought treatment, the watering was stopped immediately and continued for 15 days, and then rewatered. For salt treatment, plants were irregated with NaCl solution (300 mM) twice at a 3-day interval. All plants were photographed every two or three days. Samples used for physiological index measurements were obtained through drying treatment (withhold watering) for 10 days and salt (300 mM NaCl) for seven days, respectively.

### Estimation of water loss rate

Four-week-old plants were used for estimation of water loss rate. About 0.5 g leaves were excised from more than five plants in same status and put on filter paper on an experiment bench under condition of room temperature (~ 25 °C) and 60% relative humidity. The leaves were weighted at designed time points. Water loss rate was then calculated and three replicates for each line were performed.

### Measurements of physiological index related to stress toleance

Chlorophyll was extracted according to procedures described previously [53]. The modified acidic ninhydrin method was used to measure proline content [54]. The contents of soluble protein and soluble sugar were measured using TP Quantitative Assay Kit (Nanjing Jiancheng, Nanjing, China) and Plant Soluble Sugar Sontent Test Kit (Nanjing Jiancheng), respectively. Histochemical stains of 3, 3′-diaminobenzidine (DAB) and nitroblue tetrazolium (NBT) was used to visualize the accumulated hydrogen peroxide (H_2_O_2_) and superoxide anion radical (O_2_^−^) in leaves after stress treatment, respectively [50]. The H_2_O_2_ content as well as antisuperoxide anion activity were quantified by Hydrogen Peroxide assay kit (Nanjing Jiancheng) and Inhibition and Produce Superoxide Anion Assay Kit (Nanjing Jiancheng), respectively. Evaluation of superoxide dismutase (SOD), peroxidase (POD), and catalase (CAT) activities as well as malondialdehyde (MDA) content were conducted as described previously [55, 56]. Three replicates were executed for all these experiments.

### Analysis of stomatal aperture and endogenous ABA content

To measure stomatal movement responding to mannitol and ABA, rosette leaves of four-week-old WT and transgenic lines were floated on a solution (50 mM KCl, 0.1 mM CaCl_2_, and 10 mM MES, pH 6.15) and placed under light for 2.5 h to induce stomatal opening. Then, these leaves were transferred into the opening solutions without or with 300 mM mannitol, or 20 μM ABA, respectively, and treated in a growth incubator for a further 2 h [57–59]. Stomata on lower epidermal layers of the leaf were immediately observed and photographed by optical microscopy (DP80, Olympus, Japan). The width and length and their ratio (stomatal aperture) of more than 60 stomata for each line were measured and calculated. All experiments were repeated in three times.

Endogenous ABA content was measured by MetWare (http://www.metware.cn/) with the AB Sciex QTRAP 6500 LC-MS/MS platform. Briefly, 50 mg leaves of four-week-old WT and transgenic plants were extracted with 1 ml formic acid/water/methanol (0.5:2:7.5, V/V/V). The extract liquid was evaporated to dry in nitrogen, reconstituted with 0.1 ml 80% methanol (V/V), and then filtered for detection. Three biological replicates were conducted.

### Statistical analysis

Data generated in the present study were analyzed by the Independent-Sample T-Test in SPSS 23.0, and showed as the mean ± standard deviation (SD) of three replicates, and the significance of difference were showed as * (*P* < 0.05) and ** (*P* < 0.01).

## Data Availability

The sequence of *MfbLHL38* has been deposited in GenBank of NCBI with accession No. of MT383747 (https://www.ncbi.nlm.nih.gov/nuccore/MT383747.1/). All other data generated or analyzed during this study are included in this published article.

## Abbreviations

TFs: Transcription factors
ABA: Abscisic acid
YFP: Yellow fluorescent protein
qRT-PCR: Quantitative real-time PCR
WT: Wild-type
MDA: Malondialdehyde
ROS: Reactive oxygen species
H_2_O_2_: Hydrogen peroxide
O_2_^−^: superoxide anion radical
SOD: Superoxide dismutase
POD: Peroxidase
CAT: Catalase
